# Exposure–response relationship of ramucirumab in patients with advanced second-line colorectal cancer: exploratory analysis of the RAISE trial

**DOI:** 10.1007/s00280-017-3380-z

**Published:** 2017-07-25

**Authors:** Allen Lee Cohn, Takayuki Yoshino, Volker Heinemann, Radka Obermannova, György Bodoky, Jana Prausová, Rocio Garcia-Carbonero, Tudor Ciuleanu, Pilar Garcia-Alfonso, David C. Portnoy, Eric Van Cutsem, Kentaro Yamazaki, Philip R. Clingan, Jonathon Polikoff, Sara Lonardi, Lisa M. O’Brien, Ling Gao, Ling Yang, David Ferry, Federico Nasroulah, Josep Tabernero

**Affiliations:** 1 Rocky Mountain Cancer Center, 1800 Williams Street, Denver, CO 80218 USA; 2 National Cancer Center Hospital East, Chiba, Japan; 3 Ludwig-Maximilians-University Hospital Munich, Munich, Germany; 4 Masaryk Memorial Cancer Institute, Brno, Czech Republic; 5 Szent László Hospital, Budapest, Hungary; 6 University Hospital Motol, Prague, Czech Republic; 7 Hospital Virgen del Rocio, Seville, Spain; 8 Institutul Oncologic Cluj-Napoca, Cluj-Napoca, Romania; 9 Hospital General Universitario Gregorio Marañón, Madrid, Spain; 10 West Clinic, Memphis, TN USA; 11 University Hospitals Gasthuisberg, Louvain, Belgium; 12 Shizuoka Cancer Center, Shizuoka, Japan; 13 Southern Medical Day Care Centre, Wollongong, Australia; 14 Kaiser Permanente San Diego, San Diego, CA USA; 15 Istituto Oncologico Veneto IOV - IRCCS, Padua, Italy; 16 Eli Lilly and Company, Indianapolis, IN USA; 17 Eli Lilly and Company, Bridgewater, NJ USA; 18 Eli Lilly and Company, Buenos Aires, Argentina; 19 Vall d’Hebron University Hospital and Institute of Oncology (VHIO), Barcelona, Spain

**Keywords:** Ramucirumab, Exposure–response, Colorectal cancer, Second line, FOLFIRI

## Abstract

**Purpose:**

To characterize ramucirumab exposure–response relationships for efficacy and safety in patients with metastatic colorectal cancer (mCRC) using data from the RAISE study.

**Methods:**

Sparse pharmacokinetic samples were collected; a population pharmacokinetic analysis was conducted. Univariate and multivariate Cox proportional hazards models analyzed the relationship between predicted ramucirumab minimum trough concentration at steady state (*C*_min,ss_) and survival. Kaplan–Meier analysis was used to evaluate survival from patients in the ramucirumab plus folinic acid, 5-fluorouracil, and irinotecan (FOLFIRI) treatment arm stratified by *C*_min,ss_ quartiles (Q). An ordered categorical model analyzed the relationship between *C*_min,ss_ and safety outcomes.

**Results:**

Pharmacokinetic samples from 906 patients were included in exposure–efficacy analyses; samples from 905 patients were included in exposure–safety analyses. A significant association was identified between *C*_min,ss_ and overall survival (OS) and progression-free survival (PFS) (*p* < 0.0001 for both). This association remained significant after adjusting for baseline factors associated with OS or PFS (*p* < 0.0001 for both). Median OS was 11.5, 12.9, 16.4, and 16.7, and 12.4 months for ramucirumab *C*_min,ss_ Q1, Q2, Q3, Q4, and placebo group, respectively. Median PFS was 5.4, 4.6, 6.8, 8.5, and 5.2 months for ramucirumab *C*_min,ss_ Q1, Q2, Q3, Q4, and placebo group, respectively. The risk of Grade ≥3 neutropenia was associated with an increase in ramucirumab exposure.

**Conclusions:**

Exploratory exposure–response analyses suggested a positive relationship between efficacy and ramucirumab exposure with manageable toxicities in patients from the RAISE study with mCRC over the ranges of exposures achieved by a dose of 8 mg/kg every 2 weeks in combination with FOLFIRI.

**Electronic supplementary material:**

The online version of this article (doi:10.1007/s00280-017-3380-z) contains supplementary material, which is available to authorized users.

## Introduction

Colorectal carcinoma (CRC) is the third leading cause of cancer worldwide [1] and ranks fourth among leading causes of cancer deaths worldwide [2]. Conventional systemic therapy for CRC includes fluoropyrimidine-based regimens alone or in combination with irinotecan or oxaliplatin [3–7]. The development of agents targeting the epidermal growth factor receptor (EGFR) and angiogenic pathways has provided additional treatment options. Vascular endothelial growth factor (VEGF) and VEGF receptor-2 (VEGFR-2)-mediated signaling and angiogenesis are important in CRC tumor growth and are established therapeutic targets. Ramucirumab is a human IgG1 monoclonal antibody that specifically binds to the extracellular domain of VEGFR-2 with high affinity, preventing binding of VEGF-A, C, and D ligands and receptor activation [8]. The safety and efficacy of ramucirumab in combination with second-line folinic acid, 5 fluorouracil, and irinotecan (FOLFIRI) in patients with metastatic CRC that progressed during or after first-line therapy with bevacizumab, oxaliplatin, and a fluoropyrimidine were evaluated in a randomized, double-blind, placebo-controlled phase III trial (RAISE) [9]. On Day 1 of each 2-week cycle, patients received either 8 mg/kg ramucirumab or placebo as a 60 min intravenous infusion, followed by the FOLFIRI regimen. The RAISE trial demonstrated a statistically significant survival benefit for patients treated with ramucirumab plus FOLFIRI versus placebo plus FOLFIRI with a median overall survival (OS) of 13.3 months (95% confidence interval [CI] 12.4–14.5) for patients in the ramucirumab group versus 11.7 months (95% CI 10.8–12.7) for the placebo group (hazard ratio [HR] 0.844, 95% CI 0.730–0.976; log-rank *p* = 0.0219). Ramucirumab plus FOLFIRI was well tolerated and the adverse events were considered manageable [9].

All drugs have dose effect curves, a threshold concentration below which they are ineffective, a concentration where effect has reached a maximum plateau and between these extremes a range where increasing exposure increases effectiveness. The ‘exposure–response’ phenomenon occurs in the range of concentrations (exposure) where increasing exposure correlates with increasing effect. The phenomenon of exposure–response is seen with many antibodies in the treatment of cancer, including ipilimumab in melanoma [10], trastuzumab emtansine in breast cancer [11], rituximab in the treatment of low-grade B cell malignancies, and rilotumumab in gastric cancer [12]. Analyses of the exposure–response relationship of ramucirumab in patients with gastric cancer (REGARD and RAINBOW trials) and non-small cell lung cancer (REVEL trial) have previously been reported [13, 14]. In both RAINBOW and REGARD, higher exposure to ramucirumab was associated with longer OS and progression-free survival (PFS) for gastric cancer patients. In REVEL, higher exposure to ramucirumab was associated with longer OS and PFS for non-small cell lung cancer patients.

The objective of this exploratory analysis was to determine whether there is an exposure–response relationship for ramucirumab in patients with advanced CRC enrolled in the RAISE trial.

## Materials and methods

**Fig. 1:**
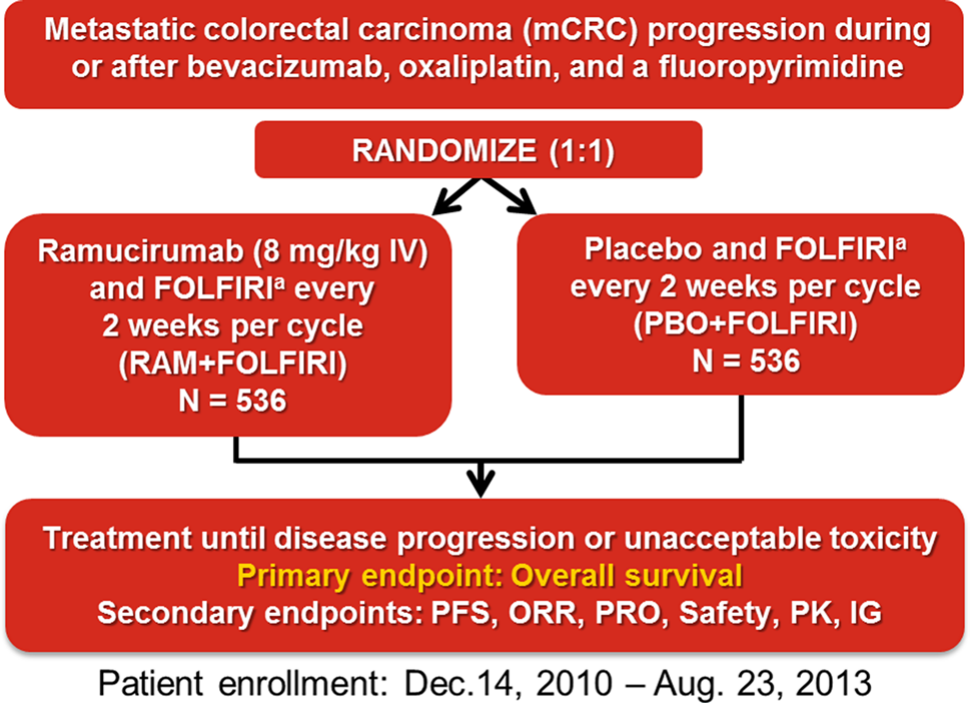
RAISE study design ^a^Irinotecan: 180 mg/m^2^; Folinic acid: 400 mg/m^2^; 5-flurouracil: 400 mg/m^2^ bolus followed by 400 mg/m^2^ given as a continuous infusion over 48 hours FOLFIRI, folinic acid, 5-fluorouracil, and irinotecan; *IG* immunogenicity; *IV* intravenous; *ORR* objective response rate; *PFS* progression-free survival; *PK* pharmacokinetics; *PRO* patient-reported outcomes; *RAM* ramucirumab

The details of the RAISE trial including patient eligibility, trial design, randomization, dose administration, clinical outcome definitions, and statistical analyses have been previously published [9]. Each center’s institutional review board or independent ethics committee approved the study. The trial followed the principles of the Declaration of Helsinki and the Good Clinical Practice Guidelines of the International Conference on Harmonisation. All patients provided written informed consent. To summarize, eligible patients included those with pathologically confirmed metastatic CRC, known KRAS exon 2 mutation status (mutant or wild-type), and an Eastern Cooperative Oncology Group performance status (ECOG PS) score of 0 or 1 with disease progression during or within 6 months of the last dose of first-line combination therapy with bevacizumab, oxaliplatin, and a fluoropyrimidine for metastatic disease. Patients were randomized (1:1) to receive on Day 1 of each 2-week cycle either 8 mg/kg ramucirumab or placebo as a 60 min intravenous infusion, followed by the FOLFIRI regimen (180 mg/m^2^ intravenous irinotecan given over 90 min followed by or concurrent with 400 mg/m^2^ intravenous leucovorin given over 120 min, followed by 400 mg/m^2^ fluorouracil given as an intravenous bolus over 2–4 min then 2400 mg/m^2^ given as a continuous infusion over 48 h; Fig. 1). Dose modifications were permitted for ramucirumab in the setting of non-life-threatening and reversible Grade 3 clinical adverse events (AEs; for example, fever) considered to be at least possibly related to investigational product and that resolved to Grade ≤1 or pre-treatment baseline within 1 treatment cycle (approximately 2 weeks). FOLFIRI modifications were allowed for each component and were based on toxicities observed and graded according to the National Cancer Institute Common Terminology Criteria for Adverse Events (NCI-CTCAE) v4.0.

The primary endpoint was OS, defined as the time from randomization to death from any cause. Key secondary endpoints included PFS (defined as time from randomization to progressive disease or death, whichever occurred first), the proportion of patients who achieved an objective response (defined as complete response or partial response), pharmacokinetic (PK) parameters of ramucirumab, and safety.

Pre-dose and 1-h post-infusion PK samples to determine ramucirumab serum concentration were collected at the following timepoints per study protocol: Day 1 of Cycles 3, 5, 9, 13, and 17. Due to the timing of PK sample collection, only patients from both treatment arms who had non-missing concentration data and who did not die or discontinue prior to Day 1 Cycle 3 were included in the exposure–response analysis. The patients included in the exposure–response analyses are a non-random subset of the intent-to-treat (ITT) population.

Serum ramucirumab concentration was determined using a validated enzyme-linked immunosorbent assay (ELISA) at Intertek Pharmaceutical Services (San Diego, CA, USA). A population PK model was developed using a nonlinear mixed-effect modeling approach (NONMEM 7.3 [Icon, Ellicott City, MD]) and in accordance with the U.S. Food and Drug Administration (FDA) Guidance for Industry on Population Pharmacokinetics [15, 16]. Population PK model-predicted minimum concentration at steady state (*C*_min,ss_) was used to assess the exposure–response relationship.

### Exposure–efficacy analysis

The exposure–efficacy analysis was conducted using SAS^®^ version 9.1.2 or higher. Univariate and multivariate Cox proportional hazards models were used to evaluate the relationship between ramucirumab exposure and efficacy endpoints (OS and PFS). Data for the ramucirumab plus FOLFIRI treatment arm were separated into four quartiles (Q) based on the exposure parameter of interest, *C*_min,ss_. Kaplan–Meier analyses were performed for OS and PFS with data from patients in the ramucirumab plus FOLFIRI arm stratified according to *C*_min,ss_ quartile; each quartile was compared with the data from patients in the placebo plus FOLFIRI (control) arm. A multivariate Cox model adjusted for baseline covariates was used to estimate the HR for each quartile versus the control arm. Stepwise Cox regression, with entry *p* value = 0.05 and exit *p* value = 0.10, selected the baseline factors prognostic for OS or PFS. These significant factors were used to adjust the final model for evaluating exposure–efficacy relationships. An additional matched case–control analysis for OS was explored to adjust for the potential imbalances in important prognostic factors between treatment arms within each exposure quartile group [17]. In this analysis, the case groups are the four exposure quartiles of *C*_min,ss_ in the ramucirumab plus FOLFIRI arm. For every patient in each case group, a matched control patient was identified from all patients receiving placebo plus FOLFIRI, through a matching scheme based on the six significant potential prognostic factors identified in the stepwise Cox regression analysis and two additional covariates with the largest baseline imbalance in the subset of patients for this analysis (sex and prior bevacizumab use—composite subgroup). The two additional factors were selected based on a stepwise logistic regression of treatment arm assignment on the same pool of covariates in the stepwise Cox regression, and stepwise selection using entry and exit *p* values of 0.2 (since randomization is supposed to make treatment assignment to be independent of baseline variables, a larger significance level was used to pick up any imbalanced factors due to chance). Missing values in any of the matching factors excluded the patients from the matched case–control study.

### Exposure–safety analysis

Ordered categorical and logistic regression models were developed to explore the relationship between ramucirumab exposure (*C*_min,ss_) and the safety endpoints. Safety endpoints for exposure–safety analysis were the three most common Grade ≥3 treatment-emergent adverse events (TEAEs) in the ITT population occurring in ≥5% of patients in the ramucirumab plus FOLFIRI arm, with a difference in incidence rate between the ramucirumab arm and the placebo arm of ≥2%. Neutropenia, hypertension, and fatigue were the selected endpoints for exposure–safety analysis based on these criteria. For this analysis, neutropenia and fatigue are consolidated terms (composite terms consisting of multiple related preferred terms) based on Standardized MedDRA^®^ Queries (SMQ) and medical review. Diarrhea was included as an adverse event of interest as it is one of the most frequent TEAE for the FOLFIRI regimen [18, 19]. Safety endpoints were graded per the NCI-CTCAE v4.0.

## Results

Data from a total of 425 patients from the ramucirumab plus FOLFIRI arm and 481 patients from the placebo plus FOLFIRI arm were included in the exposure–efficacy analyses; data from a total of 425 patients from the ramucirumab plus FOLFIRI arm and 480 patients from the placebo plus FOLFIRI arm were included in the exposure–safety analyses. One patient randomized to the placebo group received ramucirumab as the first dose and was included in the placebo plus FOLFIRI arm for exposure–efficacy analysis and excluded from the placebo plus FOLFIRI arm for exposure–safety analysis.

An exploratory Cox regression analysis identified a statistically significant positive association between OS and *C*_min,ss_ in a univariate analysis (*p* < 0.0001). A multivariate Cox regression analysis was used to adjust for the factors that were significantly associated with OS: time to progression after beginning first-line therapy, KRAS status, ECOG PS, number of metastatic sites, liver only metastasis, and carcinoembryonic antigen (CEA). After adjusting for these baseline factors, the association between OS and *C*_min,ss_ remained statistically significant (*p* < 0.0001). Similar to OS, a statistically significant positive association was identified between PFS and *C*_min,ss_ in a univariate Cox regression analysis (*p* < 0.0001). A multivariate Cox regression analysis was used to adjust for the factors that were significantly associated with PFS: ECOG PS, number of metastatic sites, liver only metastasis, CEA, and prior bevacizumab use (composite subgroup). A similar association between ramucirumab exposure and PFS was observed after adjusting for these significant baseline factors (*p* < 0.0001).

Ramucirumab concentration data from patients in the ramucirumab plus FOLFIRI arm were grouped into four *C*_min,ss_ quartiles (<25% [Q1], 25% to <50% [Q2], 50% to <75% [Q3], and ≥75% [Q4]) based on the predicted *C*_min,ss_ concentration. Demographic data and baseline disease characteristics by *C*_min,ss_ quartile are presented in Table 1. Similarity in the data in Supplemental Table 2 between the overall study population (ITT population) [9] and the exposure–response population suggests that the analyzed subpopulation is reflective of the overall population enrolled in RAISE.

**Table 1:** Exposure–response population baseline demographics and disease characteristics by ramucirumab *C*_min,ss_ quartile

	Placebo + FOLFIRI *N* = 481 *n* (%)	Ramucirumab + FOLFIRI Q1 *N* = 106 *n* (%)	Ramucirumab + FOLFIRI Q2 *N* = 106 *n* (%)	Ramucirumab + FOLFIRI Q3 *N* = 106 *n* (%)	Ramucirumab + FOLFIRI Q4 *N* = 107 *n* (%)
Age					
<65 years	287 (59.7)	70 (66.0)	63 (59.4)	67 (63.2)	65 (60.7)
≥65 years	194 (40.3)	36 (34.0)	43 (40.6)	39 (36.8)	42 (39.3)
Gender					
Female	183 (38.0)	45 (42.5)	58 (54.7)	45 (42.5)	56 (52.3)
Male	298 (62.0)	61 (57.5)	48 (45.3)	61 (57.5)	51 (47.7)
Race					
White	372 (77.3)	73 (68.9)	70 (66.0)	80 (75.5)	87 (81.3)
Asian	92 (19.1)	26 (24.5)	31 (29.2)	22 (20.8)	18 (16.8)
Other	13 (2.7)	5 (4.7)	5 (4.7)	4 (3.8)	2 (1.9)
Missing	4 (0.8)	2 (1.9)	0 (0.0)	0 (0.0)	0 (0.0)
Region					
Europe	216 (44.9)	45 (42.5)	47 (44.3)	40 (37.7)	52 (48.6)
North America	120 (24.9)	24 (22.6)	24 (22.6)	33 (31.1)	29 (27.1)
Other	145 (30.1)	37 (34.9)	35 (33.0)	33 (31.1)	26 (24.3)
ECOG PS					
0	238 (49.5)	42 (39.6)	52 (49.1)	68 (64.2)	58 (54.2)
1+	243 (50.5)	63 (59.4)	54 (50.9)	37 (34.9)	48 (44.9)
Missing	0 (0.0)	1 (0.9)	0 (0.0)	1 (0.9)	1 (0.9)
Time to disease progression after first-line therapy					
<6 months	111 (23.1)	31 (29.2)	22 (20.8)	21 (19.8)	21 (19.6)
≥6 months	370 (76.9)	75 (70.8)	84 (79.2)	85 (80.2)	86 (80.4)
*KRAS* Status					
Mutant	234 (48.6)	52 (49.1)	48 (45.3)	59 (55.7)	57 (53.3)
Wild-type	247 (51.4)	54 (50.9)	58 (54.7)	47 (44.3)	50 (46.7)
Number of metastatic sites					
1	146 (30.4)	35 (33.0)	41 (38.7)	29 (27.4)	28 (26.2)
2	177 (36.8)	40 (37.7)	40 (37.7)	42 (39.6)	45 (42.1)
≥3	158 (32.8)	31 (29.2)	25 (23.6)	35 (33.0)	34 (31.8)
Liver only metastasis					
No	394 (81.9)	86 (81.1)	82 (77.4)	92 (86.8)	91 (85.0)
Yes	87 (18.1)	20 (18.9)	24 (22.6)	14 (13.2)	16 (15.0)
Site of primary tumor					
Colon	318 (66.1)	58 (54.7)	63 (59.4)	82 (77.4)	71 (66.4)
Rectal	158 (32.8)	48 (45.3)	43 (40.6)	22 (20.8)	35 (32.7)
Colorectal	5 (1.0)	0 (0.0)	0 (0.0)	2 (1.9)	1 (0.9)
Carcinoembryonic antigen					
<200 µg/L	359 (74.6)	77 (72.6)	77 (72.6)	84 (79.2)	79 (73.8)
≥200 µg/L	91 (18.9)	23 (21.7)	23 (21.7)	16 (15.1)	21 (19.6)
Missing	31 (6.4)	6 (5.7)	6 (5.7)	6 (5.7)	7 (6.5)
Time from first bevacizumab dose to last bevacizumab dose					
<3 months	88 (18.3)	13 (12.3)	15 (14.2)	14 (13.2)	13 (12.1)
≥3 months	391 (81.3)	93 (87.7)	90 (84.9)	91 (85.8)	94 (87.9)
Missing	2 (0.4)^a^	0 (0.0)	1 (0.9)	1 (0.9)	0 (0.0)

*Note* Patients in each exposure quartile group were a non-randomized subset of the ITT population and potential imbalances in prognostic factors between the placebo arm and the quartile groups may be generated due to the loss of randomization. However, the multivariate Cox regression analysis was adjusted for all prognostic factors significantly associated with OS or PFS

*C*_min,ss_ minimum concentration at steady state, *ECOG PS* Eastern Oncology Cooperative Group performance status, *FOLFIRI* folinic acid, 5-fluorouracil, and irinotecan; *ITT* intent-to-treat, *OS* overall survival, *PFS* progression-free survival, *Q* quartile

**Fig. 2:**
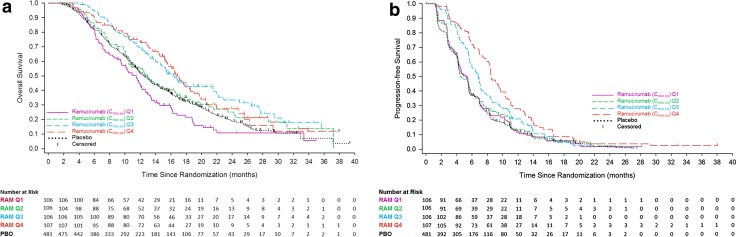
**a** RAISE overall survival by ramucirumab C_min,ss_ exposure quartile. Ramucirumab C_min,ss_ quartile concentrations: Q1 (<25%) <49.7 µg/mL, Q2 (25% to <50%) 49.7 to <62.6 µg/mL, Q3 (50% to <75%) 62.6 to <81.1 µg/mL, Q4 (≥75%) ≥81.1 µg/mL. **b** RAISE progression-free survival by ramucirumab C_min,ss_ exposure quartile. Ramucirumab C_min,ss_ quartile sconcentrations: Q1 (<25%) <49.7 µg/mL, Q2 (25% to <50%) 49.7 to <62.6 µg/mL, Q3 (50% to <75%) 62.6 to <81.1 µg/mL, Q4 (≥75%) ≥81.1 µg/mL C_min,ss_, minimum concentration at steady state; *PBO* placebo; *Q* quartile; *RAM* ramucirumab

The Kaplan–Meier plots of OS and PFS by *C*_min,ss_ quartiles are presented in Fig. 2a and b. Separation between the OS curves was observed among the four exposure groups, suggesting that higher exposure may be associated with longer OS time. Median OS was 11.5, 12.9, 16.4, and 16.7 months for the ramucirumab *C*_min,ss_ Q1, Q2, Q3, and Q4 groups, respectively (Table 2). Median OS in the placebo plus FOLFIRI arm was 12.4 months (Table 2). Hazard ratios by quartile were adjusted for baseline factors. There was a trend of numerically decreasing hazard ratios as the predicted concentration of ramucirumab *C*_min,ss_ increased. Quartile 1 and Q2 were not significantly different from the placebo group; however, Q3 and Q4 showed hazard ratios favoring the ramucirumab plus FOLFIRI treatment arm (Table 2).

**Table 2:** RAISE overall survival and progression-free survival by ramucirumab exposure quartile

Quartile, *C*_min,ss_	Overall survival^a^			Progression-free survival^b^		
	Median (mos)	Hazard ratio (95% CI)^c^	*p* value^d^	Median (mos)	Hazard ratio (95% CI)^c^	*p* value^d^
PBO + FOLFIRI	12.4^e^			5.2^e^		
RAM + FOLFIRI Q1	11.5	1.311 (1.024, 1.678)	0.0314	5.4	0.932 (0.739, 1.175)	0.5516
RAM + FOLFIRI Q2	12.9	0.954 (0.736, 1.238)	0.7249	4.6	0.957 (0.763, 1.200)	0.7013
RAM + FOLFIRI Q3	16.4	0.604 (0.459, 0.795)	0.0003	6.8	0.684 (0.542, 0.864)	0.0014
RAM + FOLFIRI Q4	16.7	0.657 (0.500, 0.865)	0.0027	8.5	0.546 (0.434, 0.688)	<0.0001

*C*_min,ss_ minimum concentration at steady state, *ECOG PS* Eastern Oncology Cooperative Group performance status, *FOLFIRI* folinic acid, 5-fluorouracil, and irinotecan, *OS* overall survival, *PBO* placebo, *PFS* progression-free survival, *Q* quartile, *RAM* ramucirumab

^a^Adjusted for time to progression after beginning first-line therapy, KRAS status, ECOG PS, number of metastatic sites, liver only metastasis, and carcinoembryonic antigen

^b^Adjusted for ECOG PS, number of metastatic sites, liver only metastasis, carcinoembryonic antigen, and prior bevacizumab use

^c^Adjusted for significant prognostic factors relative to PBO + FOLFIRI in RAISE

^d^Wald’s test of RAM quartile versus PBO + FOLFIRI

^e^Median OS and PFS for PBO + FOLFIRI differ from those reported in Tabernero et al. [9], because patients in the PBO arm who dropped out prior to the third dose were excluded from the exposure–efficacy analyses

The Kaplan–Meier plots of PFS curves were similar to those for OS (Fig. 2b). The higher exposure groups were associated with longer PFS. Median PFS was 5.4, 4.6, 6.8, and 8.5 months for the ramucirumab *C*_min,ss_ Q1, Q2, Q3, and Q4 groups, respectively (Table 2). Median PFS in the placebo plus FOLFIRI arm was 5.2 months (Table 2). The PFS hazard ratios were adjusted for baseline factors and decreased as the concentration of predicted ramucirumab *C*_min,ss_ increased by quartile. Similar to the results for OS, PFS for Q1 and Q2 were not significantly different from the placebo group, but Q3 and Q4 demonstrated progressively decreasing HRs that were significantly different from the placebo group.

A matched case–control analysis for OS was explored to evaluate the exposure–OS relationship and adjust for imbalances between the *C*_min,ss_ quartiles. As previously described, there were eight matching factors for which outcomes were to be adjusted: time to progression after beginning first-line therapy, KRAS status, ECOG PS, number of metastatic sites, liver only metastasis, CEA, gender, and combined prior bevacizumab use (composite subgroup). Patients from both ramucirumab and placebo arms were included in the exposure–response analysis only if they did not die or discontinue treatment prior to Day 1 Cycle 3 and had exposure data (*C*_min,ss_) available. The matching was performed separately for each of the four C_min,ss_ exposure quartiles (Q1–Q4) from the ramucirumab plus FOLFIRI arm (Supplemental Table 1).

To compare the two treatment arms in each of the four matched case–control groups, Kaplan–Meier curves for OS in each group are shown in Supplemental Fig. 1. Clear separation of OS curves was observed in matched Q3 and Q4, but not Q1 and Q2. As compared with matched control patients, patients in Q3 and Q4 groups showed longer survival relative to patients in Q1 and Q2 groups. This is consistent with the exposure–response association as observed earlier. In addition, the steep dose–effect relationship depicted in Supplemental Table 3 shows that the risk of death or disease progression was reduced by approximately 40% or 30%, respectively, when *C*_min,ss_ was doubled.

**Fig. 3:**
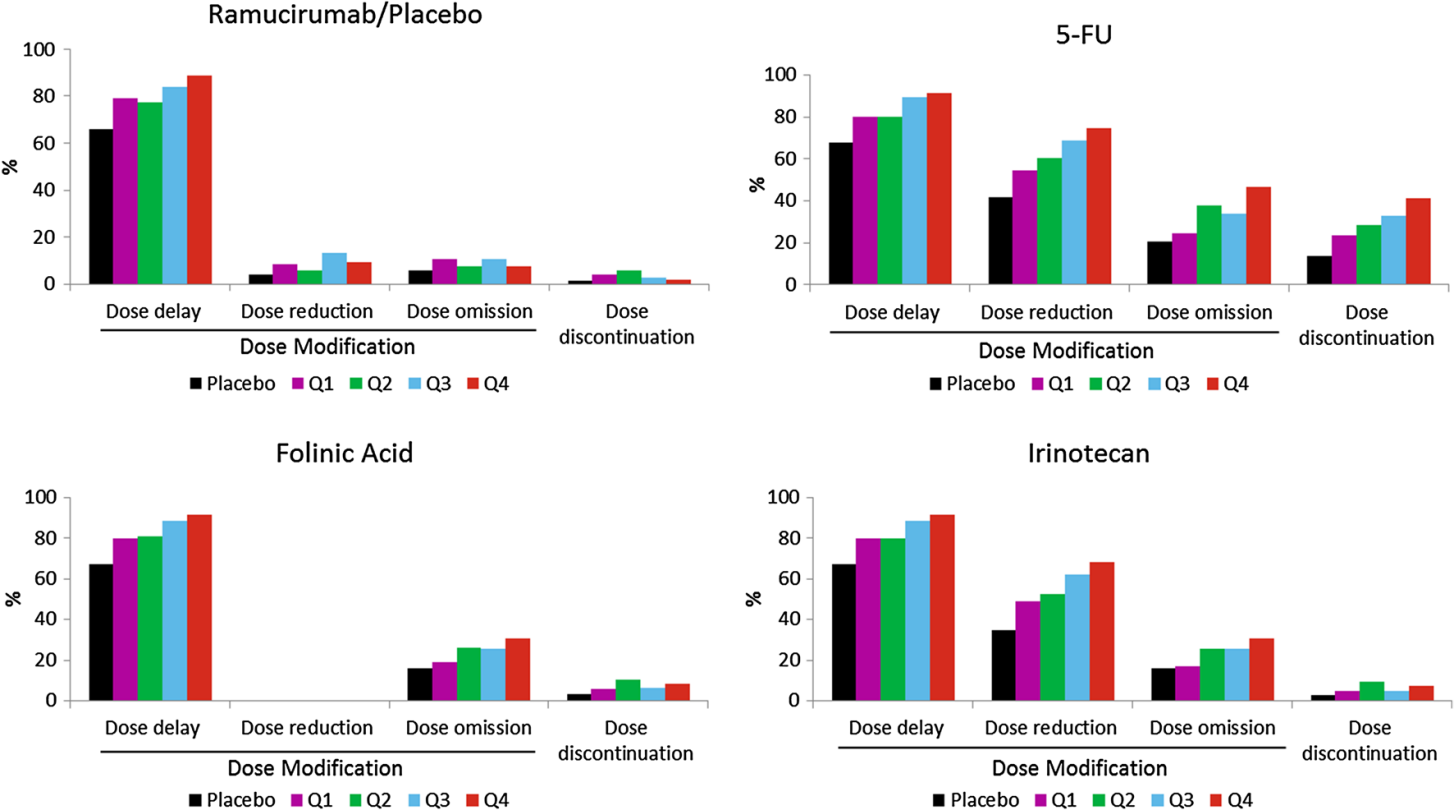
Dose omission/dose modification by exposure-response quartile 5-FU, 5-fluorouracil, *Q* quartile

The percentage of patients with dose modifications (dose delay, dose reduction, and dose omission) or dose discontinuation of ramucirumab in all four ramucirumab plus FOLFIRI quartiles was generally higher as compared to the placebo plus FOLFIRI arm (Fig. 3). Patients with higher ramucirumab exposure appeared to have higher incidence of dose delay as compared to patients with lower exposure (Fig. 3). The percentage of patients with dose modifications (dose delay, dose reduction, and dose omission) or dose discontinuation of FOLFIRI in all four ramucirumab plus FOLFIRI quartiles was generally higher as compared to the placebo plus FOLFIRI arm. In addition, patients with higher ramucirumab exposure also had higher incidence of dose modifications of FOLFIRI as compared to patients with lower exposure (Fig. 3). Higher incidence of 5-fluorouracil dose discontinuation was observed in patients with higher ramucirumab exposure. Thus, although Q4 patients had more dose reduction and delay, they still had superior PFS and OS to lower exposure quartiles.

**Fig. 4:**
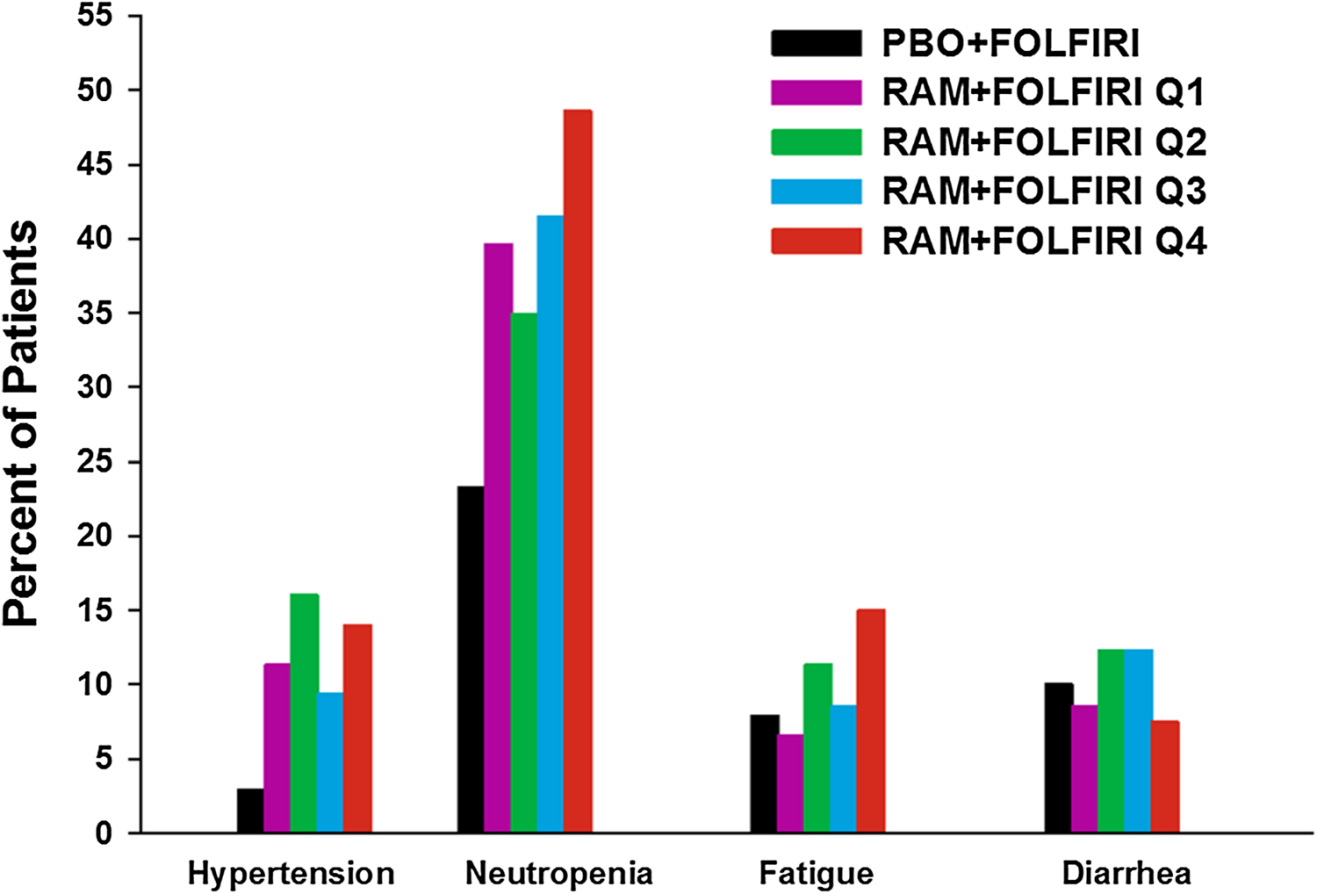
Incidence of Grade ≥3 treatment-emergent adverse events by ramucirumab C_min,ss_ exposure quartile. There was only one reported Grade 4 hypertension event, nine reported Grade 4 diarrhea events, and no Grade 4 fatigue events. A total of 9.4% patients reported Grade 4 neutropenia. There were no Grade 5 events for all four safety endpoints. Treatment-emergent adverse events were graded by NCI-CTCAE v4.0. Neutropenia and fatigue are consolidated terms, meaning they are a composite term consisting of multiple related preferred terms based on Standardized Medical Dictionary for Regulatory Activities (MedDRA) Queries and medical review. C_min,ss_, minimum concentration at steady state; FOLFIRI, folinic acid, 5-fluorouracil, and irinotecan; *NCI-CTCAE* National Cancer Institute-Common Terminology Criteria for Adverse Events; *PBO* placebo; *Q* quartile; *RAM* ramucirumab

Observed TEAE incidences (≥Grade 3) (selected safety endpoints for exposure–safety analysis) for both ramucirumab and placebo arms were similar between the overall study population and the exposure–response analysis population (Supplemental Table 2). Incidence differences between ramucirumab and placebo arms were also similar in these two populations. Therefore, the safety data used in the exposure–safety analysis were judged representative of data obtained from the overall study population. The observed incidences of Grade ≥3 neutropenia, hypertension, fatigue, and diarrhea by concentration quartile are shown in Fig. 4. There were no Grade 5 TEAEs reported. For patients receiving ramucirumab plus FOLFIRI, neutropenia was the Grade ≥3 TEAE with the highest incidence in all quartiles, and was most frequently reported in Q4, patients with the highest concentration of ramucirumab. A statistically significant (*p* < 0.001) relationship was only identified between *C*_min,ss_ and incidence of neutropenia (Grade ≥3). Severity of neutropenia (Grade 3 vs Grade 4) was independent of exposure. Additional covariate analyses found age at study entry, sex, and Asian race to be significant predictors for risk of neutropenia in CRC patients regardless of treatment arm. It is of interest to note that for neutropenia, hypertension, fatigue, and diarrhea, most of the increase in toxicity occurred between the control and Q1 and very little increase in toxicity occurred between Q1 and Q4.

## Discussion

In the RAISE trial, median OS and PFS were significantly greater for patients receiving ramucirumab plus FOLFIRI when compared to placebo plus FOLFIRI treatment [9]. Exploratory exposure–response analyses presented here demonstrate that longer median OS and PFS may be associated with increasing ramucirumab exposure as seen in patients in higher exposure quartiles (Q3 and Q4). This relationship was demonstrated with both an unadjusted analysis and a matched pair analysis. These analyses suggest that the observed exposure–efficacy relationship is independent of a patient’s baseline characteristics. Although incidence of ≥Grade 3 neutropenia was found to be significantly correlated with predicted ramucirumab concentration, severity of neutropenia (Grade 3 vs Grade 4) was independent of exposure. Grade 3 or greater febrile neutropenia in the ITT population was low (ramucirumab: 18 patients, 3%; placebo: 13 patients, 2%) [9] and could not be evaluated by quartile due to the low incidence. There was no statistically significant relationship identified between ramucirumab exposure and hypertension, fatigue, or diarrhea. Thus, the analysis suggests that some of the toxicities seem to reach maximal intensity in the Q1 population and do not get worse with increasing exposure.

Of note, for both PFS and OS in multivariate Cox regression and the matched case–control analysis, patients in the two lowest exposure quartiles had no apparent benefit from adding ramucirumab to FOLFIRI; the benefit was seen in patients with higher exposure (Q3 and Q4). This steep dose–effect relationship shows that the risk of death or disease progression was reduced when *C*_min,ss_ was doubled.

Although patients in the higher exposure quartiles appeared to have greater incidences of ramucirumab dose delay, there was no apparent relationship between ramucirumab exposure and dose reduction, dose omission, or dose discontinuation of ramucirumab. Ramucirumab dose intensity was consistent across all four quartiles, demonstrating that a ramucirumab 8 mg/kg every-2-week dosing regimen is safe. Higher incidences of dose delay, dose reduction, dose omission, or dose discontinuation of all components of FOLFIRI were observed for higher exposure quartiles. However, despite these dose reductions and delays in the Q4 group, they have superior PFS and OS compared to lower quartiles.

The development and introduction of monoclonal antibodies targeting EGFR and angiogenic pathways have expanded treatment options for cancer patients [20–30]. In most cases, the dosing strategy is based on the titration of the drug to a tolerable dose. However, clinical pharmacokinetics have been used to confirm the rationale for the recommended dose of antiangiogenic agents [31–33]. The current results with ramucirumab are also consistent with exposure–response (efficacy and safety) relationships observed for agents targeting tyrosine kinase inhibitors (TKIs). Exposure–response analyses of sunitinib, an oral multi-targeted receptor TKI, demonstrated that increased exposure is associated with longer OS, greater antitumor response, longer time to tumor progression, and some increased risk of adverse effects in patients with advanced tumors [34]. Similarly, greater *C*_min_ of nilotinib, a selective break point cluster-Abelson (BCR-ABL) TKI, was associated with shorter time to complete cytogenetic response, shorter time to major molecular response, longer time to progression, and a trend toward higher response rates in patients with chronic myeloid leukemia [35]. These results are also consistent with exposure–response relationships observed for other agents (ipilimumab, ado-trastuzumab, and rituximab) targeting pathways in cancer. A higher *C*_min_ of ipilimumab, a fully human IgG1 monoclonal antibody that blocks cytotoxic T-lymphocyte antigen-4, was associated with increased tumor responses and longer survival in patients with advanced melanoma [10]. An association between *C*_min_ levels and OS, PFS, and objective response rate was observed for ado-trastuzumab, a human epidermal growth factor 2 (HER2)–directed antibody–drug conjugate, in HER-2 positive metastatic breast cancer [11]. In addition, increased clearance of bevacizumab, a humanized monoclonal IgG1 antibody that targets VEGF-A, was also associated with poorer prognosis for gastric cancer patients [36].

Previous analyses have demonstrated that higher predicted ramucirumab exposure was associated with longer OS and PFS, smaller hazard ratios, and increased but manageable toxicity in patients with previously-treated advanced gastric or gastroesophageal cancer as well as in patients with metastatic non-small cell lung cancer [13, 14]. The ramucirumab dose of 8 mg/kg every 2 weeks regimen is a clinically effective and safe dose in the CRC indication and offers a favorable benefit-risk profile in patients with CRC. The present exposure–response analysis shows a positive relationship between efficacy and ramucirumab exposure, seen particularly in the upper two quartiles. This opens the question of whether an increased dose of ramucirumab could achieve higher exposure in more patients, thus increasing the efficacy of ramucirumab treatment while maintaining a tolerable safety profile. Such clinical trials are ongoing in gastric and gastroesophageal junction cancers: with monotherapy (NCT02443883) and in the combination therapy with paclitaxel setting (NCT02514551).

## Electronic supplementary material

Below is the link to the electronic supplementary material.

Supplementary material 1 (DOCX 197 kb)
