# Supplementary material for: Exposure–response relationship of ramucirumab in patients with advanced second-line colorectal cancer: exploratory analysis of the RAISE trial
Source: Cancer Chemother Pharmacol. 2017 Jul 25;80(3):599–608. doi: 10.1007/s00280-017-3380-z (PMC5573752; doi:10.1007/s00280-017-3380-z)
Supplement: Supplementary file 1 — Supplementary material 1 (DOCX 197 kb) [file 280_2017_3380_MOESM1_ESM.docx]

**Supplemental Fig. 1**

OS Kaplan-Meier Curves in Each Matched Case-Control Quartile. N, number of patients; OS, overall survival; Plc, placebo; Q, quartile; Ram, ramucirumab

**Supplemental Table 1**

**Baseline Factors Before and After Matching, by C_min,ss_ Quartiles**

| **Baseline Factors** | **Before Matching** | | | **After Matching** | | |
| --- | --- | --- | --- | --- | --- | --- |
|  | **Ramucirumab + FOLFIRI** | **Placebo + FOLFIRI** | **P-value** | **Ramucirumab + FOLFIRI** | **Placebo + FOLFIRI** | **P-value** |
| Q1, N | 106 | 481 |  | 105 | 105 |  |
| CEA (≥200 μg/L vs. else) | 0.22 | 0.19 | 0.50 | 0.22 | 0.21 | 1.00 |
| CEA (missing vs. else) | 0.06 | 0.06 | 1.00 | 0.06 | 0.06 | 1.00 |
| ECOG PS (0 vs. ≥1) | 0.40 | 0.50 | 0.09 | 0.40 | 0.40 | 1.00 |
| *KRAS* status (wild type vs. mutant) | 0.51 | 0.51 | 1.00 | 0.51 | 0.50 | 1.00 |
| Liver only metastasis (yes vs. no) | 0.19 | 0.18 | 0.89 | 0.19 | 0.19 | 1.00 |
| Number of metastatic sites (2 vs. else) | 0.38 | 0.37 | 0.91 | 0.37 | 0.35 | 0.89 |
| Number of metastatic sites (≥3 vs. else) | 0.29 | 0.33 | 0.49 | 0.30 | 0.32 | 0.77 |
| PD at 1^st^-line therapy (<6 vs. ≥6 months) | 0.29 | 0.23 | 0.21 | 0.29 | 0.28 | 1.00 |
| Sex (male vs. female) | 0.58 | 0.62 | 0.44 | 0.57 | 0.57 | 1.00 |
| Prior bevacizumab use (all 3 factors vs. else) | 0.75 | 0.66 | 0.11 | 0.74 | 0.76 | 0.87 |
| Q2, N | 106 | 481 |  | 106 | 106 |  |
| CEA (≥200 μg/L vs. else) | 0.22 | 0.19 | 0.50 | 0.22 | 0.22 | 1.00 |
| CEA (missing vs. else) | 0.06 | 0.06 | 1.00 | 0.06 | 0.06 | 1.00 |
| ECOG PS (0 vs. ≥1) | 0.49 | 0.50 | 1.00 | 0.49 | 0.45 | 0.68 |
| *KRAS* status (wild type vs. mutant) | 0.55 | 0.51 | 0.59 | 0.55 | 0.51 | 0.68 |
| Liver only metastasis (yes vs. no) | 0.23 | 0.18 | 0.28 | 0.23 | 0.24 | 1.00 |
| Number of metastatic sites (2 vs. else) | 0.38 | 0.37 | 0.91 | 0.38 | 0.38 | 1.00 |
| Number of metastatic sites (≥3 vs. else) | 0.24 | 0.33 | 0.06 | 0.24 | 0.23 | 1.00 |
| PD at 1^st^-line therapy (<6 vs. ≥6 months) | 0.21 | 0.23 | 0.70 | 0.21 | 0.19 | 0.86 |
| Sex (male vs. female) | 0.45 | 0.62 | 0.00 | 0.45 | 0.46 | 1.00 |
| Prior bevacizumab use (all 3 factors vs. else) | 0.72 | 0.66 | 0.30 | 0.72 | 0.75 | 0.76 |
| Q3, N | 106 | 481 |  | 105 | 105 |  |
| CEA (≥200 μg/L vs. else) | 0.15 | 0.19 | 0.41 | 0.15 | 0.13 | 0.84 |
| CEA (missing vs. else) | 0.06 | 0.06 | 1.00 | 0.06 | 0.06 | 1.00 |
| ECOG PS (0 vs. ≥1) | 0.65 | 0.50 | 0.01 | 0.65 | 0.65 | 1.00 |
| *KRAS* status (wild type vs. mutant) | 0.44 | 0.51 | 0.20 | 0.44 | 0.45 | 1.00 |
| Liver only metastasis (yes vs. no) | 0.13 | 0.18 | 0.26 | 0.13 | 0.14 | 1.00 |
| Number of metastatic sites (2 vs. else) | 0.40 | 0.37 | 0.58 | 0.39 | 0.34 | 0.57 |
| Number of metastatic sites (≥3 vs. else) | 0.33 | 0.33 | 1.00 | 0.33 | 0.37 | 0.66 |
| PD at 1^st^-line therapy (<6 vs. ≥6 months) | 0.20 | 0.23 | 0.52 | 0.20 | 0.19 | 1.00 |
| Sex (male vs. female) | 0.58 | 0.62 | 0.44 | 0.57 | 0.59 | 0.89 |
| Prior bevacizumab use (all 3 factors vs. else) | 0.68 | 0.66 | 0.73 | 0.69 | 0.66 | 0.77 |
| Q4, N | 107 | 481 |  | 106 | 106 |  |
| CEA (≥200 μg/L vs. else) | 0.20 | 0.19 | 0.89 | 0.20 | 0.18 | 0.86 |
| CEA (missing vs. else) | 0.07 | 0.06 | 1.00 | 0.06 | 0.07 | 1.00 |
| ECOG PS (0 vs. ≥1) | 0.55 | 0.50 | 0.34 | 0.55 | 0.55 | 1.00 |
| *KRAS* status (wild type vs. mutant) | 0.47 | 0.51 | 0.39 | 0.46 | 0.47 | 1.00 |
| Liver only metastasis (yes vs. no) | 0.15 | 0.18 | 0.48 | 0.15 | 0.13 | 0.84 |
| Number of metastatic sites (2 vs. else) | 0.42 | 0.37 | 0.32 | 0.42 | 0.43 | 0.89 |
| Number of metastatic sites (≥3 vs. else) | 0.32 | 0.33 | 0.91 | 0.32 | 0.32 | 1.00 |
| PD at 1^st^-line therapy (<6 vs. ≥6 months) | 0.20 | 0.23 | 0.52 | 0.19 | 0.20 | 1.00 |
| Sex (male vs. female) | 0.48 | 0.62 | 0.01 | 0.47 | 0.47 | 1.00 |
| Prior bevacizumab use (all 3 factors vs. else) | 0.66 | 0.66 | 1.00 | 0.67 | 0.75 | 0.29 |

Abbreviations: CEA, carcinoembryonic antigen; C_min,ss_, minimum concentration at steady-state; ECOG PS, Eastern Cooperative Oncology Group performance status; PD, progressive disease.

**Supplemental Table 2**

**Summary of Observed TEAE Incidence (≥ Grade 3) in Overall Study Population and Exposure-Response Analysis Population**

| Grade ≥3 | Overall Study Population | | | Exposure-Response Analysis Population | | |
| --- | --- | --- | --- | --- | --- | --- |
|  | **Ramucirumab + FOLFIRI  (N=529)** | **Placebo + FOLFIRI (N=528)** | **Incidence Difference (%)** | **Ramucirumab + FOLFIRI (N=425)** | **Placebo + FOLFIRI (N=480)** | **Incidence Difference (%)** |
|  | **n (%)** | **n (%)** |  | **n (%)** | **n (%)** |  |
| Neutropenia^a^ | 203 (38.4) | 123 (23.3) | 15.1 | 175 (41.2) | 112 (23.3) | 17.9 |
| Hypertension^b^ | 59 (11.2) | 15 (2.8) | 8.4 | 54 (12.7) | 14 (2.9) | 9.8 |
| Fatigue^c^ | 61 (11.5) | 41 (7.8) | 3.7 | 44 (10.4) | 38 (7.9) | 2.5 |
| Diarrhea^d^ | 57 (10.8) | 51 (9.7) | 1.1 | 43 (10.1) | 48 (10.0) | 0.1 |

Abbreviations: AESI, adverse event of special interest; FOLFIRI, folinic acid, 5‑fluorouracil, and irinotecan; N, total population size; n, number of patients; TEAE, treatment-emergent adverse event.

^a^Incidence of Grade ≥3 neutropenia (consolidated term).

^b^Incidence of Grade ≥3 hypertension (AESI term).

^c^Incidence of Grade 3 fatigue (consolidated term).

^d^Incidence of Grade ≥3 diarrhea (preferred term).

**Supplemental Table 3**

**Analysis of C_min,ss_ and OS and PFS for Patients Included in the Exposure-Efficacy Analysis**

| **Efficacy parameter** | **Hazard Ratio (1)  (95% CI)** | **p-Value  (Wald's)** |
| --- | --- | --- |
| Overall survival (N = 425/291 events) | | |
| Univariate analysis | 0.600 (0.490, 0.734) | <0.0001 |
| Multivariate analysis adjusting for significant factors (2) | 0.605 (0.494, 0.741) | <0.0001 |
| Progression free survival (N = 425/384 events) | | |
| Univariate analysis | 0.670 (0.564, 0.797) | <0.0001 |
| Multivariate analysis adjusting for significant factors (3) | 0.681 (0.569, 0.814) | <0.0001 |

Abbreviations: CI, confidence interval; C_min,ss,_ minimum concentration at steady-state; ECOG, Eastern Cooperative Oncology Group; HR, hazard ratio; N, number of patients; OS, overall survival; PFS, progression-free survival.

(1) With log2-transformed exposure measure, reported HR measures the change in the hazards of death when the value of C_min,ss_ is doubled.

(2) Adjusted for time to progression after beginning first-line therapy, KRAS status, ECOG performance status, number of metastatic sites, liver only metastasis, and carcinoembryonic antigen.

(3) Adjusted for ECOG performance status, number of metastatic sites, liver only metastasis, carcinoembryonic antigen, and prior bevacizumab use
